# A Time Course for Susceptibility to *Staphylococcus aureus* Respiratory Infection during Influenza in a Swine Model

**DOI:** 10.1155/2011/846910

**Published:** 2012-01-11

**Authors:** Elizabeth A. Smith, Sandeep R. P. Kumar, Jagadeeswaran Deventhiran, Thomas E. Cecere, Tanya LeRoith, Mike McGilliard, Subbiah Elankumaran, Isis Kanevsky Mullarky

**Affiliations:** ^1^Department of Dairy Science, Virginia Polytechnic Institute and State University, Blacksburg, VA 24061, USA; ^2^Department of Biomedical Sciences and Pathobiology, Virginia-Maryland Regional College of Veterinary Medicine, Blacksburg, VA 24061, USA

## Abstract

Bacterial superinfections following influenza A virus (IAV) are predominant causes of morbidity in humans. The recent emergence of methicillin-resistant *Staphylococcus aureus* (MRSA) and highly virulent IAV strains has reduced treatment options. Development of an appropriate animal model to study secondary *S. aureus* infections may provide important information regarding disease pathogenesis. Pigs are natural hosts to both IAV and *S. aureus* and have respiratory physiology and immune response comparable to humans. To establish a time course of susceptibility to *S. aureus* after IAV infection, nursery pigs infected intranasally with IAV were challenged with MRSA at different time points. Lung pathology scores and MRSA CFU were evaluated in dual-infected animals after IAV infection. Flow cytometric analysis of bronchoalveolar lavage fluid indicated differences between treatments. These results demonstrate the appropriateness of an intranasal challenge model in nursery pigs for studying the pathogenesis of IAV and *S. aureus* coinfection and provide insights into the timeframe for susceptibility of IAV-infected pigs to secondary *S. aureus* infection.

## 1. Introduction

Influenza A virus (IAV) infections in humans are generally mild and not often fatal; however, morbidity and mortality significantly increase with bacterial superinfections [1, 2]. *Staphylococcus aureus* commonly causes pneumonia in influenza patients, and methicillin-resistant strains currently account for 20–40% of all community-acquired pneumonia (CAP) [3]. The increasing prevalence of antibiotic-resistant bacteria, emergence of highly virulent IAV strains, and recent pandemics necessitate development of an appropriate animal model of polymicrobial infections to understand the pathogenesis and to identify intervention strategies.

 The majority of animal studies use adapted strains of IAV, which induce fatal respiratory disease atypical of human infection [4, 5]. Development of appropriate animal models to study the mechanisms of pathogenesis may provide important information for disease prevention, diagnosis, and treatment. Mice have traditionally been used to study IAV and bacterial coinfection. Although mice coinfected with *Streptococcus pneumoniae* and IAV suffer from more severe bronchopneumonia than mice with a single infection [6], disease synergy is not evident in the mouse model of *S. aureus* and influenza [7]. Furthermore, the IAV strains used in mouse coinfection studies are adapted to replicate effectively within the mouse, limiting the likeness of this model to human infection. Interestingly, cotton rats (*Sigmodon hispidus*) do not require IAV adaptation to succumb to infection, which induces respiratory tract lesions and clinical signs similar to humans [8]. Upon infection with IAV and *S. aureus*, the cotton rats exhibit signs of synergistic disease, with greater hypothermia, reduced recovery of pathology, and increased induction of cytokines [9].

Despite some success as polymicrobial models, rodents are limited in terms of biological and immunological similarity to humans [5, 10]. In humans, healthy individuals are generally able to recover from IAV-related illness within 7 days [11], whereas the infection in rodents is often fatal. Pigs are naturally susceptible to IAV and have been suggested as vessels for reassortment of human and avian IAV because of the presence of both types of sialic acid receptors [12, 13]. Swine are an ideal model for the study of IAV because the distribution pattern of viral attachment in respiratory tract tissues is closely mirrored to those in humans [14]. Furthermore, pigs are susceptible to *S. aureus* infection of the respiratory tract [15, 16].

Previous IAV challenge studies in humans describe the duration of viral shedding to last between 2 and 4 days after infection, during which respiratory symptoms, such as coughing and fever, peak before resolving within a week [17, 18]. IAV-infected mice are the most susceptible to secondary *S. pneumoniae* infection after the viral load peaks [19, 20]. Mice infected with *S. pneumoniae* prior to IAV infection did not differ from control, whereas secondary inoculation 3, 5, or 7 days after IAV resulted in one hundred percent mortality [20]. A successful time course for* S. aureus* and IAV infection has yet to be established in an animal model.

In this study, we demonstrated that synergistic disease occurs when IAV infection preceded *S. aureus* challenge and determined the optimal timing necessary to achieve this effect. A time course was developed to examine the susceptibility to *S. aureus* infection on day 3, 4, 5, or 6 following IAV infection in nursery pigs. Evaluation of this model confirms the suitability of the pig for study of IAV-methicillin-resistant *S. aureus* (MRSA) coinfection.

## 2. Materials and Methods

### 2.1. Infectious Agents

A recent triple reassortant cluster IV swine influenza virus (SIV) strain, A/Swine/Minnesota/1145/2007(H3N2), henceforth referred as “SIV 1145,” was grown in MDCK cells from a virus stock obtained from the Veterinary Diagnostic Laboratory, University of Minnesota. The dose of SIV 1145 was 1 × 10^7^ TCID_50_ per 1.5 mL diluted in Dulbecco's Modified Eagle's Minimum Essential Medium (DMEM). The community-acquired MRSA isolate NRS123 was obtained from the Network on Antimicrobial Resistance in *S. aureus* (NARSA) and known to carry the Panton-Valentine leukocidin (PVL) gene. To prepare bacterial inoculum, 50 mL of Trypticase soy broth (TSB) was inoculated with a single pure colony of MRSA grown overnight on Trypticase soy agar plates. The bacteria were grown at 37°C for 18 h with shaking. Bacterial pellets were washed with phosphate-buffered saline (PBS), pH 7.4, and resuspended to 1 × 10^8^ CFU per mL using absorbance at 600 nm.

### 2.2. Animals

Thirty-six conventionally reared nursery pigs, approximately 10–20 kg in bodyweight, were obtained from the swine breeding facility at Virginia Polytechnic Institute and State University, Center for Molecular Medicine and Infectious Disease animal facility, Blacksburg, VA. Animals were confirmed to be free of SIV-specific antibodies. All procedures were approved and carried out in accordance with the Institutional Animal Care and Use Committee of Virginia Tech.

### 2.3. Experimental Protocol

All pigs (*n* = 36) were acclimated for 2 days before the start of the experiment. Additionally, animals were treated for 3 days with Lincomycin at 11 mg/kg bodyweight to reduce the incumbent bacterial load prior to experimental IAV infection. Preinoculation blood samples were drawn after intramuscular injection of Tiletamine and Zolazepam (Telazol, Fort Dodge Animal Health; Forth Dodge, IA), and Xylazine (Lloyd Laboratories, Lloyd Inc. Shenandoah, IA), each at 4.4 mg/kg bodyweight. Subsequently, pigs were infected intranasally with SIV 1145 on D0 (*n* = 28). Each pig received 1.5 mL of 1 × 10^7^ TCID_50_/SIV in each nostril. Mock-infected pigs (*n* = 4) received equal volume of PBS to serve as uninoculated controls. To establish a time course of susceptibility to *S. aureus*, animals (*n* = 4 per) were challenged with 0.5 mL of 1 × 10^8^ CFU MRSA in each nostril on D3, 4, 5, or 6 following primary IAV infection and harvested 48 h following secondary infection (i.e., on D5, 6, 7, or 8). MRSA only-infected control animals (*n* = 4) also were harvested 48 h post infection. 

Animals were monitored twice daily for changes in rectal temperature and clinical score (0—no clinical signs, 1—mild clinical signs, 2—moderate clinical signs, 3—severe clinical signs). Nasal swabs were taken daily for quantification of viral shedding. Animals were humanely euthanized by the intravenous injection of sodium pentobarbital (Fatal-Plus, Vortech Pharmaceuticals; Dearborn, MI) at 0.22 mL/kg bodyweight.

Bronchoalveolar lavage (BAL) was collected immediately after euthanasia. Fifty mL of cold 0.3% EDTA in PBS was injected into the right and left anterior lobes of the lung using a sterile catheter and the fluid was aspirated immediately. Blood was collected from the anterior vena cava into EDTA tubes (BD Vacutainer; Franklin Lakes, NJ). Lung, spleen, and mediastinal lymph nodes were collected and flash frozen in liquid nitrogen and stored at −80°C until processed. Lung samples were collected in 10% neutral buffered formalin and processed for histology.

### 2.4. Bacterial and Viral Titers

Following euthanasia, lung, spleen, and mediastinal lymph nodes were stored in 20 mL of cold PBS. Homogenates were used directly for bacterial cultures. Quantification of methicillin-resistant staphylococcal colony counts was done by 10-fold dilutions on mannitol salt agar with cefoxitin (United States Pharmacopeial, Inc., Rockville, MD). Isolates were confirmed as the MRSA inoculate by positive Gram stain, by catalase and coagulase tests, and by PCR analysis for the presence of the PVL gene (data not shown). The primer sequences for the amplification of PVL gene is provided (forward primer: 5′  AGCAATGAGGTGGCCTTTC 3′, reverse primer: 5′  GGGGGTAATTCATTGTCTG 3′). Virus titers were determined from nasal swabs, blood, and lungs by 10-fold serial dilutions on MDCK cell monolayers as previously described [20].

### 2.5. Lung Pathology

Lungs were removed immediately after euthanasia and examined for gross pathologic alterations. A board-certified veterinary pathologist blinded to the experimental groups examined the lungs from each pig and scored by lobes on a severity scale; right and left caudal (1–5), right and left cranial and middle (1–10), and accessory (1–5) lobes. The overall lesion score was determined by the degree of inflammation, necrosis, and interstitial pneumonia.

### 2.6. Flow Cytometry

Flow cytometric analysis of cell surface markers in BAL was performed with monoclonal antibodies specific for porcine antigens. Fifty *μ*L of 10^6^ cell suspension were prepared in FACS wash (0.05% sodium azide in PBS) buffer. Cells were incubated with primary antibodies against CD8-*α*, CD4, 2B11, CD21, CD14, MHCII, (VMRD; Pullman, WA), and CD80 (LifeSciences, Inc.; Seattle, WA) for 1 h on ice (Table 1). Antibody combinations are provided in Table 1. The cells were washed and incubated with respective fluorochrome-conjugated isotype specific secondary antibodies (Invitrogen, USA) for 30 min on ice. The cells were washed and fixed in 100 *μ*L of 1% paraformaldehyde. Percentages of cells resulting from immunolabelling were determined using a 6-color FacsCalibur flow cytometer (BD biosciences; San Jose, CA) and analyzed using Flowjo software v7.6.1 (Tree star Inc.; Ashland, OR). Isotype controls and unstained cells were used to establish background and set appropriate gates during analysis.

### 2.7. Statistical Analysis

Data were analyzed using the Proc Glimmix procedure in SAS v.9.2 (SAS Institute Inc.; Cary, NC). *P* values were generated for paired comparisons of treatments with Tukey's tests. Significance was set to *P* < 0.05.

Analyses of lesion scores, as well as BAL cells positive for CD4, CD8, 2B11, CD21, CD80, CD14, and MHCII were conducted using Table 2. Comparisons of SIV only versus dual-infected animals were made within each harvest day. These effects in MRSA only and dual-infected animals were compared in a model that contained the effects of treatment and residual. Tukey's tests were performed to determine significant effects of treatment. 

Analyses of body temperature were conducted using Table 3. Comparisons of SIV only versus dual-infected animals were made within each harvest day.

## 3. Results

### 3.1. Polymicrobial Intranasal Challenge Model in Pigs

**Figure 1:**
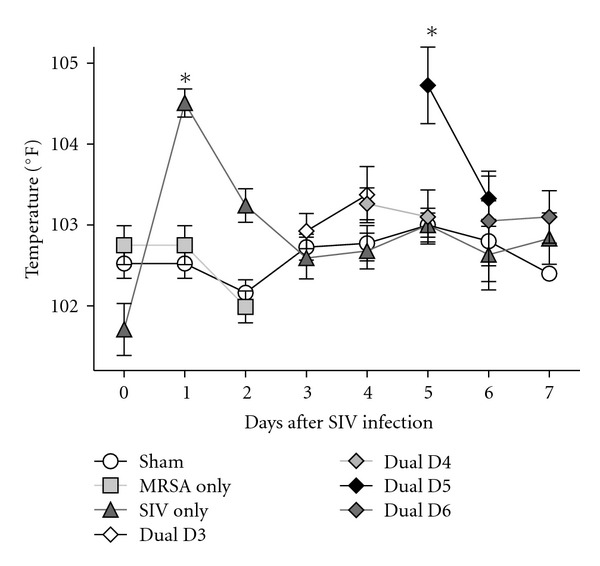
Rectal temperature during SIV and MRSA infection. Twenty-eight animals were intranasally infected on day (D) 0 with SIV 1145 (SIV only), four animals were infected with MRSA (MRSA only), and six were sham-infected with PBS (Sham). On the morning of days 3 (Dual D3), 4 (Dual D4), 5 (Dual D5), or 6 (Dual D6) following SIV infection, groups of four animals were inoculated with MRSA. Days after SIV infection are indicated on the *X*-axis, and evening body temperature is plotted on the *Y*-axis. Asterisk indicates a *P* value of <0.05 for the marked data point compared with temperature on D0. Values represent mean with standard error.

IAV infection caused significant increase in body temperature 24–48 h after infection (*P* < 0.05) (Figure 1), as well as sneezing, nasal discharge, coughing, and lethargy in infected pigs. Body temperature peaked 24–48 h after IAV infection and began to decrease, returning to normal by D3. Animals infected with MRSA on D5 after IAV infection showed a second significant increase in body temperature (*P* < 0.05) 12 h after bacterial inoculation. Body temperature for all other infected animals did not significantly differ from Sham controls. Virus shedding peaked at D3 after IAV infection (Table 4) and then decreased through D5 postinfection. No significant differences in lung PFU were detected between IAV only as compared to dual-infected animals (data not shown).

**Figure 2:**
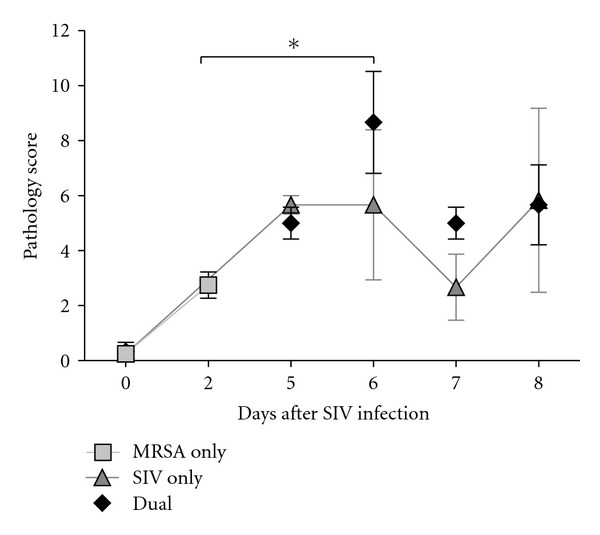
Macroscopic lung pathology in swine influenza, MRSA, and dual-infected pigs. The pathology score represents the sum of lesion scores, graded by different lobes: right and left caudal (0–5), right and left cranial and middle (0–10), and accessory (0–5) lobes. Days after SIV infection are indicated on the *X*-axis, and average score on day of harvest is depicted on the *Y*-axis. Average lesion score is depicted for MRSA only (*n* = 4), SIV only (*n* = 4), and dual-infected animals (*n* = 4). Asterisk indicates a *P* value of <0.05 for MRSA compared with animals dual-infected on D4. Values represent mean with standard error.

At necropsy, sham-infected animals showed no macroscopic signs of infection in the lung and there were no significant differences compared to pigs infected with MRSA only. However, dual-infected animals showed more disperse areas of red hepatization throughout the lung as compared with single-infected animals (data not shown) as indicated by macrosopic lung pathology scores (Figure 2). There was an overall treatment significance of *P* = 0.03. Pair-wise comparison by Tukey's tests indicated dual-infected animals inoculated with MRSA on D4 after IAV had a significantly greater lesion score compared with MRSA-only-infected animals (*P* = 0.02) at 48 h after bacterial infection.

**Figure 3:**
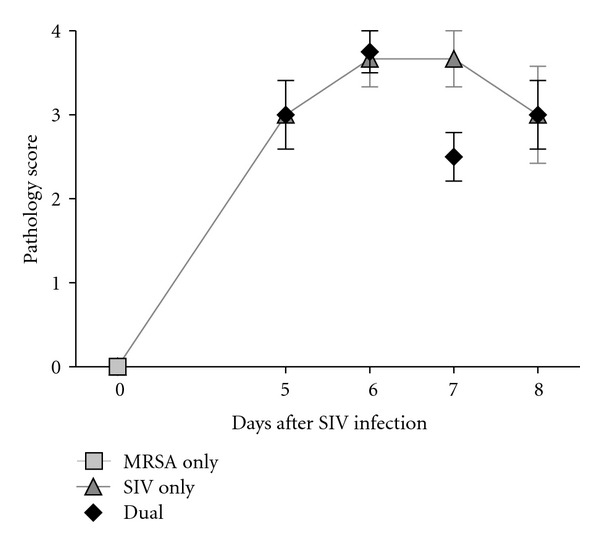
Histopathological scores of lungs in SIV- and MRSA-infected pigs. The average microscopic lung lesion scores were graded on a scale of 0 to 4 based on the severity of inflammation as mild, moderate, severe, or very severe. Average microscopic lesion score is depicted for MRSA only (*n* = 4), SIV only (*n* = 4), and dual-infected (*n* = 4) animals on multiple sections of the lung from each animal. Values represent mean with standard error.

**Figure 4:**
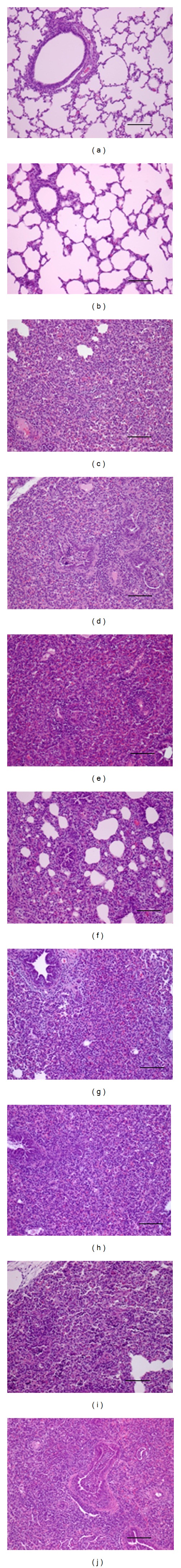
Microscopic lesions in the lung of infected pigs. (a) Sham-infected, (b) MRSA only, (c) SIV 3 days postinfection, (d) SIV 4 days postinfection, (e) SIV 5 days postinfection, (f) SIV 6 days postinfection, (g) Dual D3, (h) Dual D4, (i) Dual D5, and (j) Dual D6. Sham-infected and MRSA only groups had no microscopic evidence of pneumonia (a-b). SIV alone and Dual groups exhibited regionally extensive interstitial or bronchointerstitial pneumonia at all time points (c–j). Affected bronchioles and alveolar spaces were filled with lymphocytes, plasma cells, and fewer neutrophils. Alveolar septa were expanded by similar inflammatory cells. All bars represent 100 um.

Histopathology scores of lungs indicated an increase in the score for all infected animals. Microscopic lesion scores were significantly different over time for both IAV and dual-infected animals (*P* < 0.0001; Figure 3). There was no microscopic evidence of pneumonia in sham-inoculated (Figure 4(a)) or MRSA only (Figure 4(b)) animals. However, lungs of IAV only (Figures 4(c)–4(f)) and dual-infected (Figures 4(g)–4(j)) animals showed bronchointerstitial pneumonia and several areas with small caliber bronchioles filled with inflammatory exudate surrounded by zones of interstitial pneumonia. Bronchioles and alveolar spaces in SIV only and dual-infected animals contained lymphocytes, plasma cells, and some neutrophils.

### 3.2. SIV Infection Predisposes Pigs to Bacterial Infection

**Figure 5:**
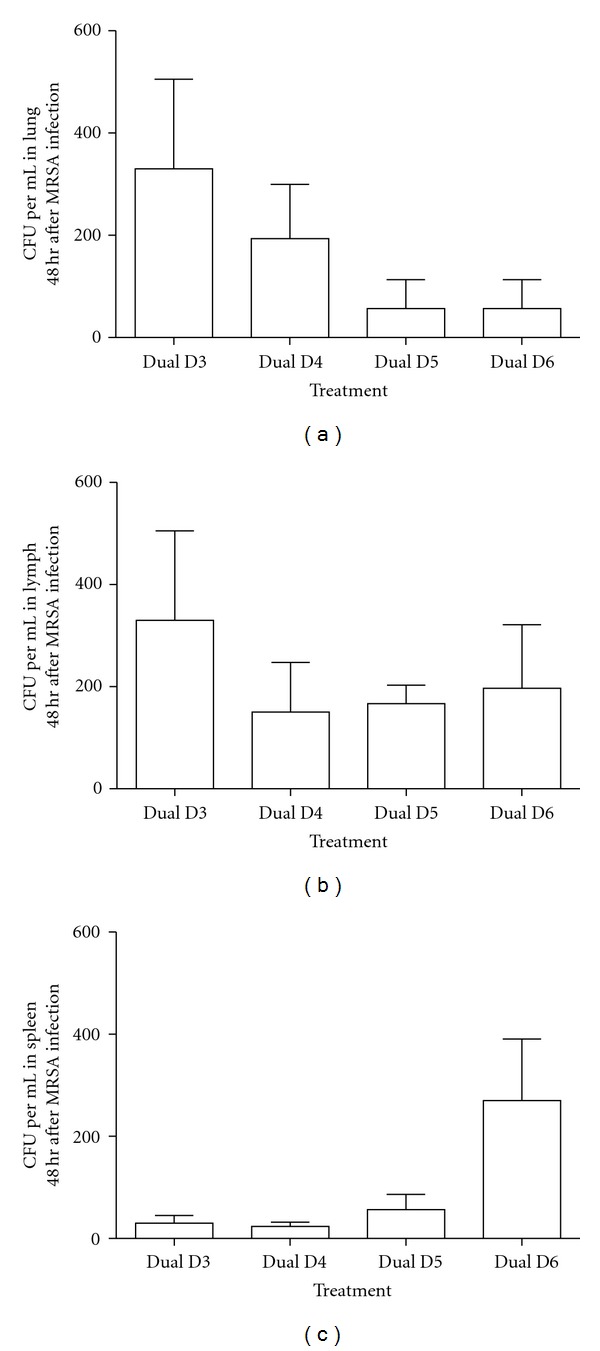
Bacterial load of MRSA in dual-infected pigs. Tissue were collected from lung (a), mediastinal lymph node (b), or spleen (c) of animals coinfected with IAV and MRSA. All tissues were homogenized, serially diluted, and plated on media selective for methicillin-resistant staphylococci. Additional biochemical tests selected for PVL + MRSA. Average CFU per mL is depicted for dual-infected animals (*n* = 4). Error bars represent the standard error.

Sham, MRSA only, and SIV only animals did not have recoverable MRSA CFU in the lung, lymph node, or spleen (data not shown). However, all animals infected with SIV had viable MRSA in the lung, lymph nodes, and spleen at 48 h after bacterial infection (Figure 5). Recoverable MRSA CFU per mL in the lung were greatest on D3, but not significantly different, and decreased steadily in dual-infected animals (Figure 5(a)). Bacterial load in the mediastinal lymph nodes did not differ by day of MRSA infection in animals with prior SIV infection (Figure 5(b)). Interestingly, spleen bacterial counts were highest in animals infected with MRSA on D6 but this was not statistically significant (Figure 5(c)).

### 3.3. Leukocyte Profiles Differ between Treatments

**Figure 6:**
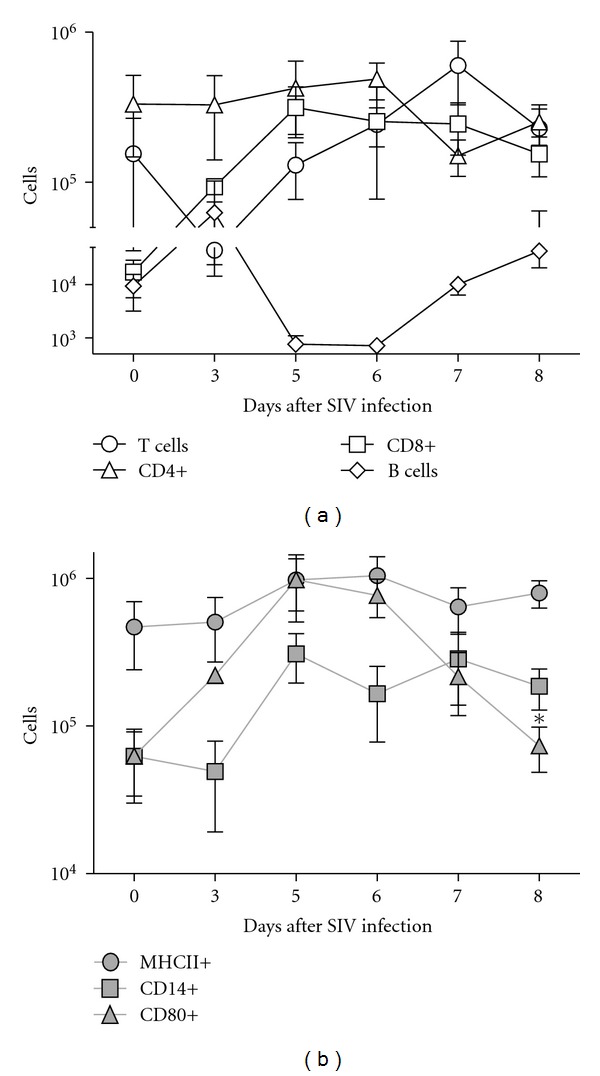
Leukocyte profiles during SIV infection. (a) Mix 1: CD4+, CD8+, 2B11+, and CD21+ (b) Mix 2: MHCII+, CD14+, and CD80+ cells within the lymphocyte gate are shown. BAL was collected from SIV-only-after infected animals on day of harvest (*n* = 3). Days after SIV-infection are indicated on the *X*-axis, and positive cells are plotted on the *Y*-axis.

**Figure 7:**
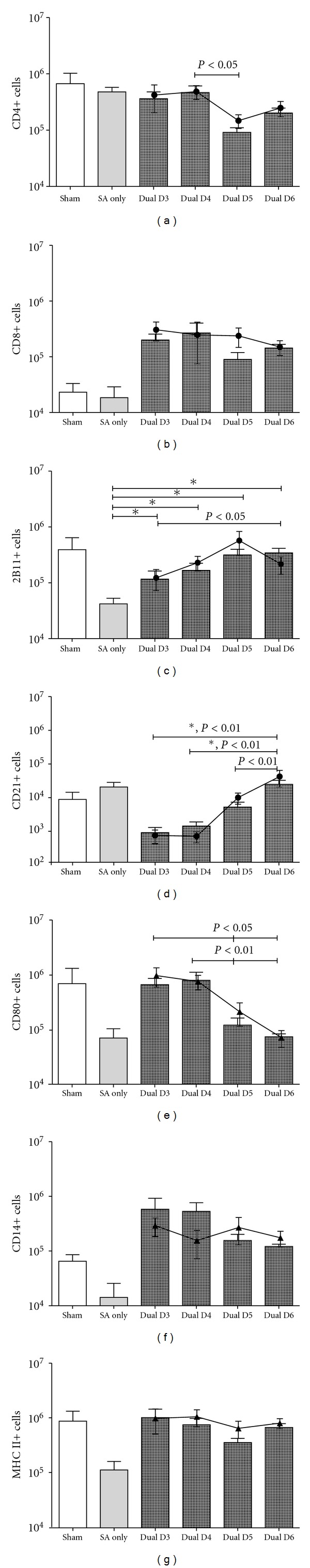
Leukocyte profiles during SIV and MRSA infection. BAL was collected from sham (*n* = 4) MRSA only, and dual-infected animals 48 h after MRSA infection (*n* = 4). The black line includes positive cells from SIV-infected animals with and without secondary infection (error bars indicate ± standard error). Treatment is indicated on the *X*-axis, and positive cells are plotted on the *Y*-axis. Cells were labeled with antibodies in Mix 1: (a) CD4+, (b) CD8+, (c) 2B11+, and (d) CD21+ or antibodies in Mix 2: (e) CD80+, (f) CD14+, and (g) MHCII+. Only cells within the lymphocyte gate are shown. There was an overall effect of treatment, with a *P* value of <0.05 for CD4+, 2B11+, CD21+ cells and CD80+ cells. Asterisks indicate a significant difference due to *S. aureus* treatment (*P* value <0.05). All other statistical information indicated difference due to SIV infection only.

BAL was collected directly following euthanasia, cells were isolated, stained, and number of lymphocytes were analyzed by flow cytometry. Leukocyte numbers from lung BAL are depicted for SIV-only-infected animals in Figure 6. In Figure 7, all SIV infected animals (single and dual depicted with black line) are compared to Sham, MRSA only, and dual-infected animals. Animals harvested on D6 after SIV infection had significantly more CD4+ cells in the lung than animals on D7 after SIV (*P* < 0.05; Figure 6(a)). However, total numbers of 2B11+ T cells were significantly greater on D8 after SIV as compared to D5 after SIV (*P* < 0.05; Figure 6(a)). Number of B cells was significantly lower on D5 and D6 after SIV as compared to D8 after SIV infection (*P* < 0.005; Figure 6(a)). Similarly, number of CD21+ B cells was lower on D7 after SIV as compared to D8 after SIV infection (*P* < 0.01; Figure 6(a)). Interestingly, there was also an increased number of CD80+ cells on D5 and D6 after SIV as compared to D7 and D8 after SIV infection (*P* < 0.05; Figure 6(b)). There were no significant differences in number of CD8+ T cells, CD14+, or MHCII+ cell types in lungs of SIV-infected animals.

There was a treatment effect on numbers of CD4+ cells when comparing MRSA only and dual-infected animals (*P* < 0.05; Figure 7(a)). The number of 2B11+ cells increased significantly in all dual-infected animals compared with MRSA-only-infected animals (*P* < 0.001; Figure 7(c)). Similarly, SIV-infected animals challenged with MRSA on D6 had a significant (*P* < 0.005) increase in the number of CD21+ cells as compared with D3 (*P* < 0.05) and D4 (*P* < 0.05, Figure 7(d)). There was a treatment effect on numbers of CD80+ cells when comparing MRSA only and dual-infected animals (*P* < 0.05; Figure 7(a)). However, the numbers of CD14+ or MHCII+ cells were not significantly affected by treatment (Figures 7(f) and 7(g), resp.). Additionally, there was no difference in the expression of any markers in dual-infected animals compared with SIV-only-infected animals.

## 4. Discussion

IAV infection establishes an environment in the respiratory tract that allows for an aggravated response to secondary bacterial infection. In this study, single infected animals did not display increased pathology compared to sham, whereas dual-infected animals had bacterial pneumonia. This study was effective in establishing the period in which IAV-infected pigs are susceptible to secondary *S. aureus* infection.

This study represents the first swine intranasal model for influenza and secondary bacterial infection. The pig possesses several similarities to human physiology and has been a successful model of human disease pathogenesis [21]. Pigs, therefore, have the potential to serve as a model for coinfection with these pathogens. Previous studies using the cotton rat model suffer from the same limitations as the mouse model in that rodents are not natural IAV hosts and they do not represent the ideal model to study the pathogenesis of lung lesions, fever, or respiratory distress. In fact, mice and cotton rats do not show an increase in body temperature, or PMN infiltration in the lungs, and the infection is frequently lethal. In this study, all pigs infected with SIV exhibited a significant rise in body temperature following inoculation compared to Sham and MRSA-only-infected animals. This is comparable with human IAV and in direct contrast with rodent models, which show a significant drop in body temperature following infection [8, 17]. Fever has been correlated with PMN infiltration into the lung, as well as with IFN-*α*, TNF-*α*, IL-1, and IL-6 production in response to SIV infection [11]. Following viral clearance, the anti-inflammatory cytokine, IL-10, is produced to reduce inflammation. Previous research suggests that the secretion of IL-10 due to IAV infection predisposes mice to secondary bacterial infection due to the inhibitory effects of this cytokine [1]. We are currently investigating whether this may in fact be the case in the porcine model.

Lung lesion scores were greatest in IAV-infected animals inoculated with MRSA on D4 (Figure 2), directly following the resolution of fever. However, MRSA was unable to establish an infection without prior SIV infection. MRSA appears to capitalize on a state of predisposition created by primary SIV infection. This is evident by the bacterial load in the lungs of dual-infected animals, when compared with MRSA-only-infected animals that had no recoverable CFU. The fact that MRSA was unable to manifest an infection without prior SIV infection, apparent by the lack of pathology, is consistent with previous polymicrobial models [20]. A previous study indicated that the influenza viral hemagglutinin (HA) protein enhanced *S. aureus* internalization by human epithelial cell lines [22]. Furthermore, influenza results in impaired NK cell function [23] and macrophage phagocytic function [24] that is most likely responsible for the increased *S. aureus* growth in IAV-infected lungs. Furthermore, the detectable MRSA CFU in both lymph and spleen tissue is indicative of bacterial dissemination as seen in both humans as well as the cotton rat dual infection model [9].

The increase in pathology of dual-infected animals compared to single and mock infected animals may be due to several factors. MRSA is capable of causing tissue damage during infection, thereby contributing to the exacerbated damage of lung parenchyma in dual infected animals compared with those only infected with SIV. It is also plausible that the increase in pathology in dual-infected animals is due to effector functions of immune cells. PMN and alveolar macrophages (AMs) are credited with inducing cell damage within lungs infected with IAV and *S. pneumoniae*, by the extensive release of proinflammatory cytokine [25]. The high bacterial load within the lung of IAV-infected animals inoculated with MRSA on D3 may be responsible for the increased number of lung T and B cells. Previous studies have indicated an important role for lung cytokines in controlling lymphocyte function during dual infections. Coinfection of mice with H1N1 IAV and *S. aureus* resulted in increased type I and type II IFNs but decreased IL-17, 22, and 23 at 24–48 h after bacterial infection. The same study indicated that overexpression of IL-23 rescued cytokine production and reduced bacterial load [26]. In our study, *S. aureus* infection had a significant effect on the overall number of 2B11+ T cells, within the lung. However, numbers of CD4+ or CD8+ cells were not affected. This suggests that other subtypes of T cells might be affected by infection. One such subtype, T_h_17 cells, has been shown to be affected by IAV [27]. Further investigations into dendritic cell function, cytokine production, and subsequent T cell profiles are required to understand the immune responses during polymicrobial infections.

Interestingly, the similarity in immune cell profiles between dual-infected and SIV-only-infected animals suggests that the immune response is primarily dictated by SIV. Previous research has suggested that innate immune cell impairment during IAV infection creates a predisposed state, optimal for secondary bacterial infection [20, 28, 29]. The analysis of proinflammatory cytokines within the lung will help to explain the increased pathology of dual-infected animals and explain possible differences in immune cell function.

These results highlight the days directly following fever resolution as when pigs are susceptible to *S. aureus* infiltration. The Center for Disease Control and Prevention suggests that those presenting Influenza-like symptoms should remain home for at least 24 hours after fever resolution, without fever-reducing medication [30]. This recommendation is supported by this study, as SIV-infected pigs were susceptible to secondary MRSA 1-2 days after fever resolution, and 3-4 days after SIV infection.

## Figures and Tables

**Table 1:** List of antibodies used for flow cytometry. All primary antibodies are mouse antiporcine.

Antibody	Mix	Specificity	Source	Clone	Isotype/target isotype
Primary	1	CD8	VMRD	PT81B	IgG2b
Primary	1	CD4	VMRD	IL-A11	IgG2a
Primary	1	2B11	VMRD	2B11	IgM
Primary	1	CD21	VMRD	GB25A	IgG1
Primary	1	MHCII	VMRD	BAQ150A	IgG3
Primary	2	CD14	VMRD	(M-M9) CAM36A	IgG1
Primary	2	MHCII	VMRD	TH16B	IgG2a
Primary	2	CD80	LifeSpan	LS-C12059	IgM

Secondary	1	PE goat anti-mouse	Invitrogen	M32404	IgG2b
Secondary	1	PE-CY5.5 goat anti-mouse	Invitrogen	M32218	IgG2a
Secondary	1	AlexaFluor 594 goat anti-mouse	Invitrogen	A-21155	IgG3
Secondary	1, 2	FITC goat anti-mouse	Santa Cruz	sc2082	IgM
Secondary	1, 2	APC goat anti-mouse	Invitrogen	A10541	IgG1
Secondary	2	PE goat anti-mouse	eBioscience	12-4210-82	IgG2a

**Table 2:** 

Effect	df	F/R	ddfm
Type	1	F	20
Harvest day	3	F	20
Type ∗ Harvest day	3	F	20
Residual	20	R	

**Table 3:** 

Effect	df	F/R	ddfm
Treatment	1	F	5
Pig (treatment)	5	R	
Day	7	F	35
Treatment ∗ day	7	F	35
Residual	35	R	

**Table 4:** Virus shedding determined from daily nasal swabs of animals inoculated with SIV 1145 on day 0. Average PFU/mL and standard error of the mean (SEM) are presented for number (*n*) of pigs infected.

Day after SIV	PFU/mL	SEM	*n*
1	6.6	2.9	10
2	63.2	27.8	10
3	299.1	244.7	10
4	63.9	24.7	7
5	7.7	3.4	5
